# The Sense of Coherence as a Mediator of the Negative Effect of Discrimination on the Quality of Life in the Migrant Population

**DOI:** 10.3390/healthcare13040366

**Published:** 2025-02-08

**Authors:** Alfonso Urzúa, Diego Henríquez, Sara Hernández, Alejandra Caqueo-Urízar

**Affiliations:** 1 Escuela de Psicología, Universidad Católica del Norte, Antofagasta 1270709, Chile; sara.hernandez@alumnos.ucn.cl; 2 Centro de Justicia Educacional CJE, Pontificia Universidad Católica de Chile, Santiago 7820436, Chile; xdiegohenriquez@gmail.com; 3 Instituto de Alta Investigación, Universidad de Tarapacá, Arica 1000000, Chile; acaqueo@academicos.uta.cl

**Keywords:** quality of life, sense of coherence, discrimination, migration, salutogenesis

## Abstract

Migration generates changes in the quality of life (QoL) of immigrants. One of the difficulties that the process of migrating can entail is discrimination, which is the set of negative attitudes towards a person, in this case, because of their nationality. **Objective:** To analyze the effect that the sense of coherence (SOC) has on the relationship that discrimination by national origin has on quality of life. **Method:** The WHOQOL-BREF questionnaire was used to assess QoL, and Krieger’s Perceived Discrimination Experiences scale and Antonovsky’s SOC-13 scale were applied to 2144 participants (49.9% women), aged between 18 and 82 years, from Venezuela, Colombia and Peru, all of them being first generation migrants living in Chile. **Results:** In all the national groups evaluated, discrimination has an inverse relationship with both QoL and SOC, while the latter has a positive relationship with QoL. The direct effect of discrimination on QoL life is found to be smaller when this relationship is mediated by the presence of the SOC. This proposed model has good goodness-of-fit indicators in the three national groups evaluated. **Conclusions:** A strong and well-defined life purpose, a perception of greater control over surrounding events and situations, and perceiving life events as understandable and consistent can be an effective tools to diminish the effect of discrimination on quality of life in the migrant population.

## 1. Introduction

Migration is the movement of people away from their usual place of residence, either across an international border or within a country, whether voluntary or forced [1]. The number of immigrants worldwide reached 281 million in 2020, representing 3.6% of the world population [2]. In Latin America and the Caribbean, the migratory flow has also increased, being the place of residence of approximately 15 million immigrants in 2020 [3]. In Chile, the migratory flow has had a large increase in recent years, currently representing 8.8% of the total population [4], with the main migratory majorities coming from Venezuela (33.8%), Peru (15.9%), Colombia (12.02%), Haiti (11.72%), and Bolivia (9.39%) [5].

Migration generates changes in the lives of immigrants, which can have negative or positive effects on health, whether physical or mental, and on their well-being and quality of life (QoL). The World Health Organization defines QoL as an individual’s perception of their place in existence, in the context of the culture and value system in which they live, and in relation to their goals, expectations, norms, and concerns [6]. Operationally, it can be described as the state or feeling of well-being derived from both the objective and subjective evaluation of the degree of satisfaction of the person in different dimensions of their life. Studies that inquire about the factors that affect QoL in the migrant population are scarce [7,8,9,10,11]. Our research group has presented evidence that in the south–south migrant population living in Chile, the variables associated with QoL are age, monthly income level, who they live with, whether they are in a couple, whether the maintenance of customs from the country of origin prevails, the presence of anxiety and depression, collective self-esteem, and ethnic identity [12,13,14].

Although there are not many studies, there is evidence in the non-migrant population that quality of life can be affected when a person feels discriminated against [15,16].

Discrimination is a set of negative attitudes, such as distinction, exclusion, restriction, or other differential treatments of a person because of their religion, nationality, sexual orientation, among others, which have the intention and/or effect of impairing the recognition and exercise of human rights and freedoms in the political, economic, social, and cultural spheres [17,18]. There are not many studies that show how discrimination can affect the QoL of people who migrate, either internally or internationally, but all of them indicate the negative effect it has on QoL [19,20,21,22,23,24,25,26]. In the south–south migrant population, we have found only one study exploring this relationship [27], but, considering the regional evidence of the negative effect of discrimination on indicators like quality of life such as well-being and life satisfaction [28,29,30,31,32], we hypothesize that this negative effect would be reproduced similarly to studies conducted in other parts of the world.

There is evidence that the presence of various personal resources makes it possible to confront discrimination, change the meaning it has in their lives, and thus be able to buffer its negative impact. Some of these resources are resilience [33], autonomous orientation and integration strategies [34], perceived functionality of one’s race [35], perceived social support [36], ethnic identity [32], self-esteem [29], positive affect [31], and sense of coherence [37].

Sense of coherence (SOC) is the attitude according to which individuals—in this case, immigrants—understand life. In addition, it is a measure of the ability to evaluate and use available resources to manage the stressors faced in life—in this case, discrimination. SOC is composed of three factors: comprehensibility, understood as the ability to perceive life events as understandable and consistent and to reasonably predict what will happen in the future; manageability, which is the ability to understand that the resources at one’s disposal are sufficient to cope with life’s difficulties; and finally, meaningfulness, which is the degree to which one feels that life is emotionally meaningful [38]. In the migrant population, it has been reported that the SOC could influence the academic performance of national and international students in their first year at universities [39] or could act as a moderator of acculturative stress and a predictor of psychological well-being in Pakistani immigrants [36,40].

Regarding the relationship between discrimination and the SOC, evidence of the SOC as a buffer for the negative effects of discrimination on health in various populations can be found in the literature [41,42,43], as can evidence of it acting as a moderator in the relationship between racial discrimination and oral health-related quality of life in preschoolers [44].

In the present study, we have chosen national origin (being Venezuelan, Peruvian, or Colombian) as the discrimination variable, since, in previous studies, we have reported that this type of discrimination has a greater effect than racial discrimination, given by phenotype, on health outcomes [45].

Based on the above evidence, it is possible to appreciate the existence of studies on dual relationships: (1) discrimination and QoL, (2) SOC and QoL, and (3) SOC and discrimination. However, no literature has been found that explores the triad discrimination, SOC, and QoL in the immigrant population, so the objective of this research is to analyze the possible mediating effect of the SOC on the national origin discrimination–QoL relationship. We hypothesized that the direct effect of discrimination on QoL is lower when this relationship is mediated by the presence of the SOC.

## 2. Materials and Methods

### 2.1. Participants

The questionnaires were completed by 2144 migrants from Peru (31.3%), Colombia (32.6%), and Venezuela (36.1%). Of these, 1075 were men (50.1%) and 1069 women (49.9%) between 18 and 82 years of age, with a mean age of 34.4 years (SD = 10.7). The questionnaires were collected in five cities in Chile, with a total of 492 people completing them in the city of Santiago (22.9%), 452 in Antofagasta (21.1%), 450 in Arica (21%), 450 in Temuco (21%), and 300 in Concepción (14%). Inclusion criteria were as follows: a first-generation migrant of Venezuelan, Colombian, or Peruvian origin, a resident in Chile for more than 3 months, and over 18 years of age.

### 2.2. Measures

#### 2.2.1. Sense of Coherence

The Spanish version of the Life Orientation Scale proposed by Antonovsky [46], in its 13-item format (SOC-13), was used to assess this variable. This scale, with a Likert-type response format with scores ranging from always (1) to never (7), is composed of three dimensions: comprehensibility, manageability, and meaningfulness. This scale has reported good psychometric indicators in different Latin American populations [47,48], as well as in Peruvian [49], Venezuelan [50], and Colombian [51] populations. Given the unequal number of items that make up each factor, for the purposes of statistical analysis and comparison, the average of each factor was used instead of the sum of the raw scores.

#### 2.2.2. Quality of Life

This variable was measured using the Spanish version of the WHOQOL-BREF questionnaire [52,53], developed by the World Health Organization [54]. This instrument is made up of one question that inquiries about the general evaluation of QoL, another about satisfaction with health, and 24 others grouped in four domains: physical, psychological, social relations, and environmental, all of them with a Likert scale response format ranging from “Very bad/Very dissatisfied/Not at all/Never” = 0 to “Totally/Very good/Very satisfied/Extremely/Totally” = 4. For interpretation purposes, the scores are then transformed in each dimension to a scale ranging from 4 to 20 points. This questionnaire has shown adequate psychometric properties in the Latin American population [55], including Colombian [56] and Peruvian [57].

#### 2.2.3. Perceived Discrimination

The Perceived Discrimination Experiences Scale of Krieger et al. [58] was used. It consists of 9 items, which assess perceived discrimination for belonging to their country of origin (Colombia, Peru, or Venezuela) in 9 different contexts, such as school, work, housing, access to public services, in the health system, and by the police, among others. This scale has been used previously in studies with the Latin American migrant population [45]. For analytical purposes, the average of the items that make up the scale was used.

### 2.3. Procedures

This research is part of a larger project investigating salutogenic factors in a migrant population, which was approved by the Ethics Committee of the Universidad Católica del Norte. Using a non-representative type of sampling, combining snowball and selective sampling, participants were contacted through various places with a large migrant population, such as the Jesuit Migrant Service, the Department of Foreigners and Migration, health centers, workplaces, and neighborhoods with a high number of migrant inhabitants. Each participant signed a consent form before participating in the research.

The data were then entered into a database in SPSS.v24. First, descriptive analyses were carried out for each of the variables incorporated in the model. In the second stage, simple mediation analyses were carried out using four different models using structural equations. In all models, the mediating role of the sense of coherence in the relationship between discrimination and quality of life was assessed. The first model (M1) integrated the three samples together (Colombian, Peruvian, and Venezuelan migrants). The second model (M2) focused exclusively on the Colombian sample. The third model (M3) analyzed only the Peruvian sample. Finally, the fourth model (M4) focused exclusively on the sample of Venezuelans. In all models, the effect of sex and age on the endogenous variables was controlled for.

Model fit was assessed using the Chi-square indices (χ^2^), root mean square error of approximation (RMSEA), comparative fit index (CFI), and Tucker–Lewis index (TLI). According to the standards recommended in the literature [59], values of RMSEA ≤ 0.08, CFI ≥ 0.95, and TLI ≥ 0.95 are considered adequate, indicating a good fit. The robust maximum likelihood (MLR) method was used for all estimations, which is robust to the assumption of multivariate normality [60]. The analysis of the database was performed using the statistical packages SPSS version 24 and Mplus version 8.9.

## 3. Results

### 3.1. Sense of Coherence

Table 1 shows the means and standard deviations of each of the dimensions that make up the SOC-13 scale, both at the level of the total sample and separated by country and sex. As can be seen, both at the level of the total sample and by country, the dimension with the highest mean was meaningfulness, while the lowest was manageability. There were no statistically significant differences by sex between the means of men and women at both the country and total sample levels in any of the dimensions evaluated. Similarly, no statistically significant differences were observed by country of origin.

When analyzing the relationship between age and the various dimensions, it was found that, in the total sample, age was directly related to the domains of manageability (r = 0.090; *p* = 0.000), comprehensibility (r = 0.086; *p* = 0.000), and total score (r = 0.081; *p* = 0.000).

### 3.2. Quality of Life

Table 2 shows the means and standard deviations of each of the domains that make up the WHOQOL-BREF Quality of Life Questionnaire, both at the level of the total sample and separated by country and sex. As can be seen, both at the level of the overall sample and in the different samples, the best evaluated domain was the physical domain, while the worst evaluated domain was the environmental domain. However, statistically significant differences by country of origin were only observed in the mean of the physical QoL dimension (F(2, 2134) = 6.982; *p* = 0.001), where the mean of Venezuela was significantly higher than that of Peru (*p* = 0.001), and in the mean of the psychological QoL (F(2, 2135) = 5.295; *p* = 0.005), where the mean of Venezuela was also significantly higher than that of Peru (*p* = 0.004).

In the total sample, statistically significant differences were observed in the means between men and women for the domains of physical health (t = 2.763(2135); *p* = 0.006) and environmental quality of life (t(2122) = 2.068; *p* = 0.039); in both cases, the mean of men is significantly higher than that of women. When analyzed by country, significant differences were only found in the Peruvian sample, where the mean of men is significantly higher than that of women in the psychological QoL domain (t(666) = 2.260, *p* = 0.024); *p* = 0.024), and in the Venezuelan sample in the physical QoL domain (t(769) = 3.662; *p* = 0.000) and in the psychological QoL domain (t(770) = 2.119; *p* = 0.034), with the mean of men being significantly higher than that of women in both domains.

When analyzing the relationship between age and the various domains, it was found that, in the total sample, age was directly related to the psychological (r = 0.080; *p* ≤ 0.000) and environmental (r = 0.048; *p* = 0.027) domains. When stratifying the analysis by country, it was found that age was significantly related only to the psychological domain in Colombia (r = 0.121; *p* = 0.001) and Venezuela (r = 0.087; *p* = 0.015).

### 3.3. Discrimination

Table 3 shows the means and standard deviations of the Krieger Perceived Experiences of Discrimination scale, both at the level of the total sample and separated by country and sex. No differences by country of origin are observed. At the level of the total sample, statistically significant differences are observed between the means of men and women (t(2142) = 2.867; *p* = 0.004), where the mean of men is higher than that of women, which is repeated only in the sample from Peru (t(670) = 4.229; *p* = 0.000). At the level of the total sample, age correlates significantly and inversely with perceived discrimination (r = −0.066; *p* = 0.002), a fact that is repeated only in the Colombian sample (r = −0.126; *p* = 0.001).

### 3.4. Mediation Model

Table 4 shows the goodness-of-fit indicators of the estimated models. All four models presented adequate goodness-of-fit indices [61]. This indicates that all models are an adequate representation of the observed relationships.

**Figure 1:**
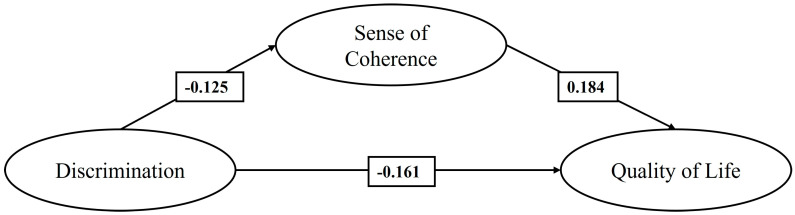
Mediating effect of the SOC on the relationship between discrimination and QoL of Colombian, Peruvian, and Venezuelan migrants (M1).

Figure 1 shows M1, which shows that discrimination has a negative effect, statistically significant and of slight magnitude (>0.10) [61], on the sense of coherence and quality of life. In turn, the sense of coherence shows a positive effect, also statistically significant and of slight magnitude, on quality of life. The indirect effect of the sense of coherence on the relationship between discrimination and quality of life was negative and statistically significant (−0.023, *p* < 0.001).

**Figure 2:**
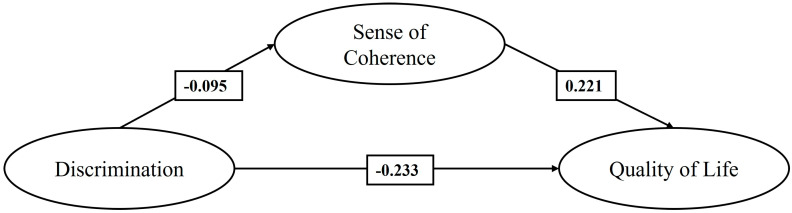
Mediating effect of the SOC on the relationship between discrimination and QoL of Colombian migrants (M2).

Once M1 was estimated, considering the three groups jointly, we proceeded to analyze the model separately for each group. Figure 2 shows M2, focused exclusively on the sample of Colombian migrants. In this model, it is observed that discrimination exerts a statistically significant negative effect, with a magnitude close to slight, on the SOC and, with a small magnitude (>0.20), on QoL [61]. Likewise, the sense of coherence has a positive effect, also statistically significant and of small magnitude, on QoL. In addition, the indirect effect of the SOC on the relationship between discrimination and QoL was found to be negative and statistically significant (−0.021, *p* < 0.001).

**Figure 3:**
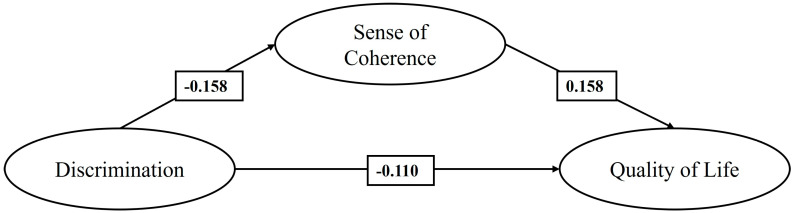
Mediating effect of the SOC on the relationship between discrimination and QoL of Peruvian migrants (M3).

In M3 (Figure 3), which considers only the sample of Peruvian migrants, it is observed that discrimination has a negative effect, statistically significant and of slight magnitude, on both the SOC and QoL. Similarly, the SOC has a positive effect, also statistically significant and of slight magnitude, on QoL. Additionally, the indirect effect of the SOC on the relationship between discrimination and QoL is negative and statistically significant (−0.025, *p* < 0.001).

**Figure 4:**
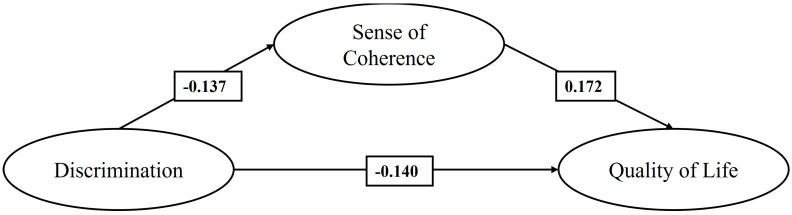
Mediating effect of the SOC on the relationship between discrimination and QoL of Venezuelan migrants (M4).

Finally, in M4 (Figure 4), which analyzes only the sample of Venezuelan migrants, it is observed that discrimination has a negative effect, statistically significant and of slight magnitude, on the SOC and QoL. At the same time, the sense of coherence has a positive effect, statistically significant and of slight magnitude, on QoL. Finally, the SOC presents a negative and statistically significant indirect effect on the relationship between discrimination and QoL (−0.024, *p* < 0.001).

## 4. Discussion

The objective of the present study was to analyze the possible mediating effect of the SOC on the national origin discrimination–QoL relationship, hypothesizing that the direct effect of discrimination on QoL is lower when this relationship is mediated by the presence of the SOC.

In relation to the SOC, it was found that the dimension that presented the highest mean, in all groups of immigrants, was meaningfulness, which indicates that people find meaning and purpose in life, while considering that the challenges they face are worth their effort [62]. This is consistent with the fact of migrating, given that the main causes of migration are related to the objectives of improving levels of well-being (in all its dimensions), making this a main objective towards which all the actions of people who migrate are directed. On the other hand, the dimension with the lowest mean was manageability, associated with the degree to which access to the necessary resources to face the demands of the environment is perceived [62]. This indicates that the migrant population evaluated, regardless of their nationality, perceives a lower availability of available resources under their control, independent of their management capacity, which is also to be expected from the migratory condition, especially in first-generation migrants, where they are still building their support networks and their situation will depend heavily on external variables (migration policies, labor conditions, support networks, migration status, housing conditions, etc.).

In relation to the QoL variable, as expected, the best evaluated dimension is the physical dimension, which can be explained by the fact that this is a young group, with a higher probability of having good physical health and of perceiving themselves as pain-free, with good functionality, and with mobility and work capabilities, as well as being able to carry out their daily activities without difficulties. On the other hand, the worst evaluated domain is the environmental domain, which is related to the perception of fewer economic resources, fewer opportunities for information, leisure, or rest, and a worse physical environment (place where they live), among others, which has also been reported in the migrant population. Men presented higher means than women in the three countries in the four domains studied, which could be associated with the Latin American sociocultural context and the gender stereotypes experienced by women and men, where the latter are positioned as providers of the household and are alien to the expression of their emotional needs, vulnerability, and request for help, which is why they tend to report higher quality of life indices than women [63,64]. Regarding the relationship between age and quality of life, we have found that older age is related to higher levels in the psychological dimension, which could be due to the fact that with age, it would be possible to access or develop a wider range of coping resources, greater adaptation, a different sense of life, or other psychological variables that could generate mechanisms and tools that affect their perception of QoL.

When analyzing the discrimination variable, the present study found that men perceive themselves to be more discriminated against than women, a phenomenon that could be explained by the Latin American social context and the stereotypes that men experience when facing processes that affect them in a negative way [63,65,66].

In general terms, the results continue to provide evidence of the negative effect of discrimination on quality of life, where the perception of higher levels of discrimination is associated with a worse evaluation of QoL, a fact that is repeated in the three migrant populations studied, but which, as already mentioned, has also been reported in Anglo-Saxon and Asian studies, with internal and international migrants [19,20,21,22,23,24,25,26,27]. This relationship could be explained by various phenomena experienced by the migrant population, such as the stress of being discriminated against and the effects on health [45,67], the relationship between discrimination and self-esteem [30], the importance of social support networks in coping with discrimination [68,69], and its long-term effects.

Regarding the effect of the sense of coherence on the relationship between national origin discrimination and QoL, we found evidence that the SOC, independent of the presence of other variables such as sex and age, which were controlled, as well as the migrant’s country of origin, presents a partial mediating effect, explaining the presence, in all groups, of a lower effect of discrimination on quality of life. This makes sense considering that the SOC, in the context of Antonovsky’s salutogenic model [38], constitutes, together with general resistance resources, a buffering factor of stress, in this case, caused by discrimination, through the way in which the person faces the world. Thus, in this context, having a strong and well-defined life purpose (meaningfulness), having a perception of greater control over surrounding events and situations (manageability), and perceiving life events as understandable and consistent, allowing one to reasonably predict what will happen in the future, will be an effective tool to diminish the effect of discrimination on quality of life. It should be noted that, as already mentioned, this protective effect of the SOC on the inverse relationship between discrimination and various health indicators is already beginning to be reported in several studies [41,42,43], this being the first study focusing on south–south migrants.

Although the diversity of factors that can influence quality of life is recognized, the present study makes a significant contribution to understanding the negative effects of perceived discrimination on QoL in the south–south migrant population, but mainly to highlighting possible protective factors that could mitigate this effect. However, some limitations should be contemplated. The present study was conducted under a non-experimental cross-sectional design, so it should be noted as a limitation that under this type of design, it is only possible to infer temporal associations and not causality, so we suggest conducting longitudinal studies to study the causes and effects over time of migrants in the face of discrimination and their QoL. Unfortunately, the complex and variable population dynamics of the migrant population makes it impossible to define this universally and to have representative samples of this human group, so any method used of a non-representative type could involve biases, and, even when the techniques used introduce elements that attempt to reduce them, the participants will not necessarily be representative of the general universe. In addition, it is suggested that the sample be expanded to include migrants from other south–south countries, Central America, and other continents, as this will allow a better understanding of the phenomenon with the similarities and differences between the different samples. It is also suggested to study the effects of discrimination in the QoL in first-, second-, and third-generation migrants, in which it will be possible to study and know how the effects of discrimination act in the different generations focusing on the age variable. Finally, although in this study, we have prioritized the variables age and sex to control the analyses, since they are the ones that most report differences in the three variables under study, it is necessary to make the model more complex in the future by incorporating other variables that could have an effect on the relationship between the variables. Examples of this could be legal status, employment conditions, and social support networks, which, in future model complexifications, could have a control effect, or even a moderating effect, as could the level of social support.

Even though the results are similar in the three national groups studied, we believe it is important to continue deepening the findings of this study, developing, for example, comprehensive studies that allow us to incorporate cultural differences in the construction and experience of the variables studied; for example, exploring the difference of discrimination towards an entire group, as in this study towards a national group, in a cultural context where an individualistic culture prevails, and in relation to other Latin American contexts, where a more collectivist culture may prevail, as well as the effects that this may have on the collective or on the individuals belonging to these collectives. The same could be applied to research on the sense of coherence, which could also be constructed differently depending on the national group. Evaluating these possible cultural or identity-based differences, as well as the incorporation of other variables in more complex models, as we have mentioned, can allow for better targeting of public policies aimed at these differences and the development of interventions based on intersectionality.

## 5. Conclusions

We consider it relevant to highlight that, although there is no evidence in the reviewed literature on experimental studies on the SOC and QoL dyad, the results found seem to suggest that the intentional modification of the SOC may be a good predictor of changes in people’s quality of life, since it would allow them to realize or resignify the impact that the way they perceive the world around them may have on their levels of well-being. This last point is relevant, given the multivariate nature of the factors that determine people’s well-being and quality of life, where a single factor, as in this case the sense of coherence, can only explain part of the variation in the relationship between discrimination and quality of life, since this interacts with multiple psychological, social, cultural, educational, and other factors. Nevertheless, for practical purposes, a small variation in the SOC can mean changes in a person’s quality of life that could even have cumulative effects in the long term. In summary, these results provide evidence of the need to consider variables such as manageability, meaningfulness, and comprehensibility in the implementation of field interventions in the migrant population, since they constitute real coping strategies that will strengthen the well-being and quality of life of migrants.

## Figures and Tables

**Table 1:** Means (X) and standard deviation (SD) of SOC-13 (score 1–7).

			MEA		MAN		COM		Total	
			X	SD	X	SD	X	SD	X	SD
Colombia	M	350	4.54	0.98	4.24	0.98	4.45	1.07	4.41	0.83
	F	348	4.55	0.86	4.18	0.97	4.37	1.09	4.37	0.76
	TOT	698	4.55	0.92	4.21	0.97	4.41	1.08	4.39	0.79
Perú	M	337	4.63	0.93	4.27	1.02	4.47	1.06	4.46	0.84
	F	335	4.48	0.93	4.18	0.97	4.36	1.07	4.34	0.82
	TOT	672	4.55	0.93	4.23	0.99	4.42	1.06	4.40	0.83
Venezuela	M	388	4.49	0.92	4.19	0.99	4.44	1.07	4.38	0.81
	F	386	4.58	0.94	4.20	1.07	4.37	1.13	4.38	0.85
	TOT	774	4.54	0.93	4.20	1.03	4.40	1.10	4.38	0.83
Total	M	1075	4.55	0.94	4.23	1.00	4.45	1.07	4.41	0.83
	F	1069	4.55	0.91	4.19	1.00	4.37	1.1	4.37	0.81
	TOT	2144	4.54	0.92	4.21	1.00	4.41	1.08	4.39	0.82

MEA = meaningfulness; MAN = manageability; COM = comprehensibility; M = male; F = female; TOT = total.

**Table 2:** Means (X) and standard deviation (SD) of WHOQOL-BREF (score 4–20).

			PHY		PSY		REL		ENV	
			X	SD	X	SD	X	SD	X	SD
Colombia	M	350	14.93	2.63	14.23	2.71	14.13	3.14	13.78	2.60
	F	348	15.02	2.65	14.55	2.76	14.14	3.46	13.58	2.69
	TOT	698	14.97	2.64	14.39	2.74	14.13	3.30	13.68	2.65
Perú	M	335	14.81	2.54	14.39	2.59	14.10	3.10	13.80	2.54
	F	334	14.51	2.33	13.96	2.41	14.05	2.76	13.53	2.32
	TOT	669	14.66	2.44	14.17	2.51	14.07	2.93	13.66	2.43
Venezuela	M	388	15.51	2.49	14.85	2.75	14.41	3.27	13.87	2.55
	F	385	14.82	2.72	14.42	2.91	14.21	3.28	13.65	2.48
	TOT	773	15.16	2.63	14.64	2.84	14.31	3.27	13.76	2.52
Total	M	1073	15.10	2.57	14.50	2.70	14.22	3.18	13.82	2.56
	F	1067	14.79	2.59	14.32	2.72	14.14	3.19	13.59	2.50
	TOT	2140	14.94	2.58	14.41	2.71	14.18	3.18	13.70	2.53

PHY = physical domain; PSY = psychological domain; REL = social relationships domain; ENV = environmental domain; M = male; F = female.

**Table 3:** Means (X) and standard deviation (SD) of discrimination for national origin Scale (score 0–3).

			X	SD
Colombia	M	350	1.51	0.35
	F	348	1.51	0.34
	TOT	698	1.51	0.64
Peru	M	337	1.55	0.67
	F	335	1.36	0.49
	TOT	672	1.45	0.59
Venezuela	M	388	1.53	0.69
	F	386	1.48	0.63
	TOT	774	1.51	0.66
Total	M	1075	1.53	0.67
	F	1069	1.45	0.60
	TOT	2144	1.49	0.63

M = male; F = female.

**Table 4:** Goodness-of-fit indicators for the models.

Models	Parameters	χ^2^	gl	*p*	CFI	TLI	RMSEA	RMSEA IC 90%	
								Low	Sup
M1	145	2341.770	340	0.000	0.954	0.949	0.052	0.050	0.054
M2	145	1042.164	340	0.000	0.947	0.941	0.054	0.051	0.058
M3	145	1061.550	340	0.000	0.944	0.938	0.056	0.052	0.060
M4	145	915.408	340	0.000	0.967	0.964	0.047	0.043	0.050

## Data Availability

Data will be made available upon request to the corresponding author.
